# Supplementary material for: MetaRibo-Seq measures translation in microbiomes
Source: Nat Commun. 2020 Jun 29;11:3268. doi: 10.1038/s41467-020-17081-z (PMC7324362; doi:10.1038/s41467-020-17081-z)
Supplement: Supplementary file 10 — Supplementary Data 7 [file 41467_2020_17081_MOESM10_ESM.zip › File2/Confidence_High_Taxonomy/377657_out.krona.html]

Javascript must be enabled to view this page.

members
magnitude
magnitudeUnassigned
count
unassigned
taxon
rank

377657\_out

99

superkingdom
99
2

976
99
phylum

200643
99
class

order
171549
99

815
99
family

99
816
genus

species
47678
99

SRS011302\_contig\_number\_25434SRS012273\_contig\_number\_34002SRS013476\_contig\_number\_30881SRS013521\_contig\_number\_4437SRS014313\_contig\_number\_1105SRS014412\_contig\_number\_13141SRS014459\_contig\_number\_19165SRS014613\_contig\_number\_11786SRS014683\_contig\_number\_12531SRS014923\_contig\_number\_46526SRS015065\_contig\_number\_6276SRS015264\_contig\_number\_9730SRS015431\_contig\_number\_18066SRS015782\_contig\_number\_26776SRS017433\_contig\_number\_3244SRS017701\_contig\_number\_2809SRS018049\_contig\_number\_111SRS018133\_contig\_number\_2758SRS018427\_contig\_number\_2014SRS018623\_contig\_number\_3069SRS018836\_contig\_number\_20718SRS019030\_contig\_number\_5124SRS019161\_contig\_number\_7262SRS019267\_contig\_number\_5226SRS019910\_contig\_number\_2173SRS020328\_contig\_number\_20936SRS020394\_contig\_number\_10429SRS022071\_contig\_number\_32722SRS022137\_contig\_number\_11243SRS024132\_contig\_number\_37541SRS024492\_contig\_number\_15028SRS042966\_contig\_number\_20101SRS043667\_contig\_number\_2230SRS043768\_contig\_number\_15472SRS043841\_contig\_number\_contig-100\_174.63629SRS044535\_contig\_number\_1709SRS045826\_contig\_number\_6977SRS046068\_contig\_number\_1374SRS047044\_contig\_number\_14785SRS048164\_contig\_number\_2930SRS049773\_contig\_number\_17773SRS049995\_contig\_number\_17279SRS050299\_contig\_number\_9900SRS050422\_contig\_number\_11886SRS050998\_contig\_number\_13308SRS051882\_contig\_number\_2209SRS052078\_contig\_number\_152SRS054905\_contig\_number\_25532SRS058723\_contig\_number\_2913SRS058770\_contig\_number\_4237SRS063518\_contig\_number\_6656SRS064276\_contig\_number\_17013SRS064557\_contig\_number\_2311SRS064757\_contig\_number\_16095SRS065176\_contig\_number\_9115SRS065504\_contig\_number\_4110SRS075878\_contig\_number\_15716SRS075984\_contig\_number\_16307SRS077024\_contig\_number\_10863SRS077086\_contig\_number\_11112SRS077335\_contig\_number\_4220SRS077454\_contig\_number\_1477SRS077502\_contig\_number\_8459SRS077589\_contig\_number\_6960SRS078176\_contig\_number\_12017SRS078242\_contig\_number\_22758SRS1041090\_contig\_number\_7341SRS1041091\_contig\_number\_15148SRS1041092\_contig\_number\_13957SRS1041118\_contig\_number\_16663SRS1041141\_contig\_number\_9241SRS104311\_contig\_number\_20034SRS104327\_contig\_number\_1405SRS1055049\_contig\_number\_13662SRS1055076\_contig\_number\_1326SRS142542\_contig\_number\_803SRS142599\_contig\_number\_16789SRS142781\_contig\_number\_1542SRS143181\_contig\_number\_10478SRS143342\_contig\_number\_254SRS143523\_contig\_number\_15677SRS143722\_contig\_number\_14590SRS144183\_contig\_number\_29930SRS144362\_contig\_number\_15301SRS147039\_contig\_number\_3040SRS147557\_contig\_number\_25412SRS147614\_contig\_number\_2324SRS147977\_contig\_number\_4695SRS148091\_contig\_number\_10800SRS148196\_contig\_number\_21832SRS148253\_contig\_number\_8418SRS148424\_contig\_number\_22876SRS148817\_contig\_number\_12048SRS149244\_contig\_number\_968SRS149784\_contig\_number\_26512SRS893279\_contig\_number\_11762SRS893285\_contig\_number\_1163SRS893327\_contig\_number\_contig-100\_783.67981SRS893378\_contig\_number\_20979
